# Early Career Scientists’ Guide to the Red Blood Cell – Don’t Panic!

**DOI:** 10.3389/fphys.2020.00588

**Published:** 2020-07-29

**Authors:** Anna Bogdanova, Lars Kaestner

**Affiliations:** ^1^Red Blood Cell Research Group, Institute of Veterinary Physiology, Vetsuisse Faculty and the Zurich Center for Integrative Human Physiology (ZIHP), University of Zurich, Zurich, Switzerland; ^2^Theoretical Medicine and Biosciences, Saarland University, Homburg, Germany; ^3^Experimental Physics, Saarland University, Saarbrücken, Germany

**Keywords:** red blood cell, senescence, Clearance, adaptation, Comparative, function, morphology

## Abstract

Why should we take interest in studying red blood cells? This mini review attempts to answer this question and highlights the problems that authors find most appealing in this dynamic research area. It addresses the early career scientists who are just starting their independent journey and facing tough times. Despite unlimited access to information, the exponential development of computational and intellectual powers, and the seemingly endless possibilities open to talented and ambitious early career researchers, they soon realize that the pressure of imminent competition for financial support is hard. They have to hit deadlines, produce data, publish, report, teach, manage, lead groups, and remain loving family members at the same time. Are these countless hardships worth it? We think they are. Despite centuries of research, red blood cells remain a mysterious and fascinating study objects. These cells bring together experts within the family of the European Red Cell Society and beyond. We all share our joy for the unknown and excitement in understanding how red cells function and what they tell us about the microenvironments and macroenvironments they live in. This review is an invitation to our colleagues to join us on our quest.

## With Inspiration and in Memory of Douglas Adams and His Ultimate Hitchhiker’s Guide to the Galaxy (Adams, 1996)

Red blood cells have power over us, no doubt. Making up over 50% of our cells (2 × 10^13^ cells), these cells provide us with energy to live, think, and create (Bianconi et al., 2013; Lew and Tiffert, 2017). Each day, we lose 1.7 × 10^11^ cells and make the same number anew (Lew and Tiffert, 2017). However, the deeper we delve into the red blood cell universe, the humbler we feel, as we still have no ultimate answer to “the Question of Life, the Universe, and Everything.”

Are all the 1.7 × 10^11^ cells we produced today the same? Do they differ from the cells we made yesterday when we went hiking in the mountains or were swimming in the lake? How do the heat waves associated with climate change affect these cells? How does microgravity affect them when, e.g., hitchhiking through the galaxy? How do the cells change as we get older and older with the increasing life expectancy?

Do the red cells produced today pass away on the same day and from the same cause? For humans, the causes of death and lifespan of individual cells seem to be somewhat random (Kaestner and Minetti, 2017), and the investigations to be done are complex, as we cannot trace the life cycle of each individual cell back as detectives do. We do not know exactly what forces red blood cells to die and how they exactly cease to exist. Some of us acknowledge oxidation-induced clustering of band 3 proteins in the membrane as a signal that tags cells with antibodies against these clusters as “labeled for removal” (Lutz, 2012; Lutz and Bogdanova, 2013). Others describe red cell death as an “apoptosis-like” process and call it eryptosis (Lang et al., 2006, 2008). The “Nomenclature Committee on Cell Death,” specializing in terminology, warns us for translation of the process as well as the word “eryptosis” to the way the enucleated cells pass away (Galluzzi et al., 2018). Is there one or more than one way to die? This question remains unclear and must still be resolved.

**FIGURE 1:**
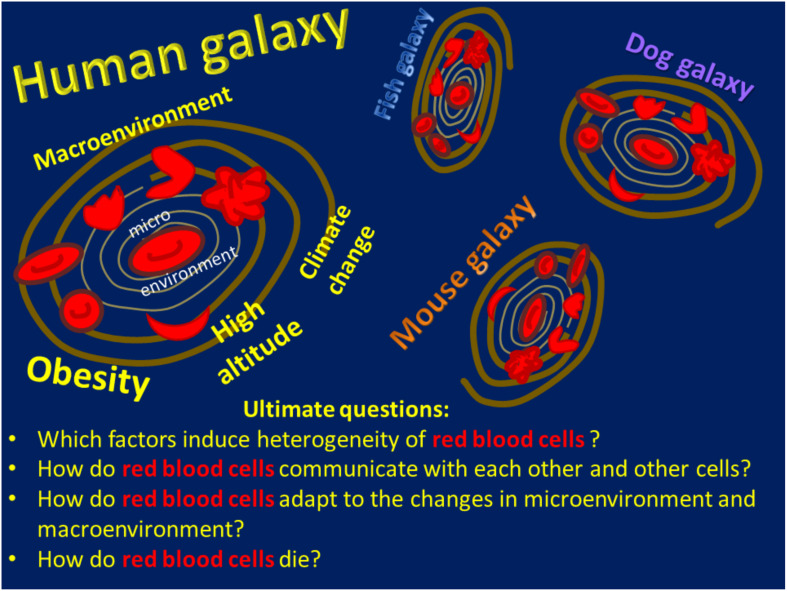
A schematic of the Red Blood Cell Universe as the authors see it, including a list of ultimate questions.

What do we need to learn about red blood cells? As in Adams’ novels, experts and early career scientists witness dynamic developments in the field that leave us both excited and thrilled. We seem to know much about the major function of red blood cells, which is gas transport, but there is much more to what these cells are doing. This makes it difficult to limit the number of ultimate questions to just one (Figure 1).

There are more than 100 years of evidence for the active participation of red blood cells in blood coagulation (Duke, 1983; Andrews and Low, 1999; Steffen et al., 2011; Byrnes and Wolberg, 2017). This concept, however, did not mature enough to enter the textbooks. How many million years will it take?

There are some indications that red blood cells may sense the changes in plasma levels of hormones, such as insulin (Pedersen et al., 1982; Zhang et al., 2015), catecholamines (Hasan et al., 2017) and cortisol (Farese and Plager, 1962), sex hormones (Koefoed and Brahm, 1994), and erythropoietin (Trial et al., 2001; Mihov et al., 2009). If so, what happens to the cells as hormones interact with the receptors on the red blood cell membrane or cytosolic components?

**FIGURE 2:**
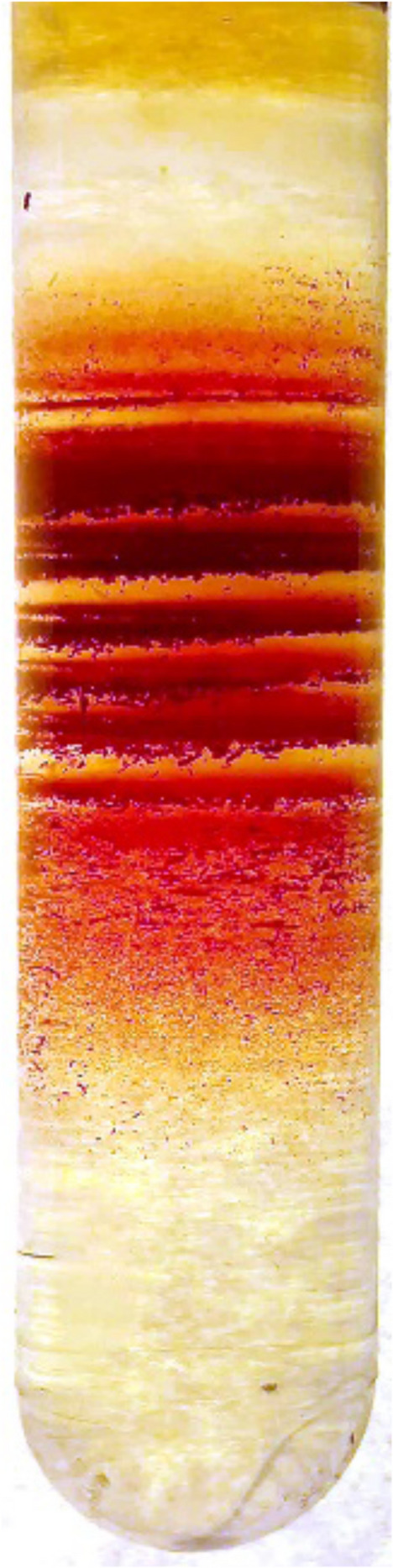
Red blood cells may have different densities, as shown on this continuous Percoll density gradient, but some densities are favored, and others are mysteriously abandoned.

Red blood cells are famous because they are widely used as a perfect cell model for studying cell membranes (Agre and Parker, 1989; Bernhardt and Ellory, 2003; Yawata, 2003). Earlier, all cells looked the same to the observers, and their properties were studied “en masse” e.g. using radioactive isotopes, rubbing cells between two plates to examine their viscosity and deformability, by inflating or deflating them, and by applying all possible approaches to whole blood samples. Mean volume and hemoglobin content values were assigned to them. Only desperate experts, such as Marcel Bessis, were photographing red blood cells for their beauty and turning their appearance into art by means of scanning electron microscopy (Bessis, 1974). If we want to study cells of similar densities and, eventually, ages, we may apply centrifugal force to produce fractions of such cells (Figure 2). Everyone that has once done such an experiment appreciates that each red cell has a certain density and joins one, but not the other group of cells of certain density, producing a striped pattern in centrifuged samples. Why certain densities are favored and others avoided is a question that needs answering. Our current understanding of red blood cell shapes includes their individual appearance and properties, their dynamic shape transitions, and their “shape memory” (Fischer, 2004; Tomaiuolo, 2014; Lanotte et al., 2016; Cordasco and Bagchi, 2017; Kihm et al., 2018). These studies make use of cellular and molecular biophysics, sophisticated *in vivo* imaging techniques for microfluidic channels and even blood vessels, and a great deal of artificial intelligence – even coming close to “Deep Thought,” the supercomputer in Adams’ story – but there is one crucial difference: patients do not have a million years to wait for the answer.

Most red blood cell researchers are deeply devoted to developing novel tools that may reveal so far unnoticed sides of cells we all like to study. Benefits are include protein profiling of red blood cells using omics approaches (Nemkov et al., 2018). Technologies use stem cells for production and manipulation of red blood cells (Hansen et al., 2019). We may follow red blood cells running through the blood vessels of living hosts (Hertz et al., 2019; Slovinski et al., 2019) and model and evaluate responses of red blood cells to mechanical shear forces – the ones they are exposed to in our microvasculature (Lizarralde Iragorri et al., 2018; Moura et al., 2019). We can detect electric currents that ions mediate passing through red blood cell membranes in hundreds of individual cells at the same time (Rotordam et al., 2019). As progress in research is fast, sometimes careful examination of the possible pitfalls and sources of artifacts is required (Minetti et al., 2013).

There is even more to explore in the universe of pathophysiology. For some patients, we do not have an answer as to what causes a red blood cell defect and only know the symptoms. Even when the molecular cause of the disease is clear, the links between the defective protein (a mismatch in amino acid composition or a dysregulated production program) and disease severity are often unknown. This is the case for sickle cell disease, hereditary spherocytosis, and Gardos channelopathy, just to name a few examples. Some patients get scientists involved into a thrilling quest for the actual cause of disease, those that were identified by hematologists as carriers of “idiopathic hemolytic anemia.” New tools are currently in development that will enable the diagnosis of “newly identified” diseases (Kaestner and Bianchi, 2020).

Even more mysterious cases are described when defects in red blood cells come along with neurodegenerative symptoms. One such disease was named “acanthocytosis” (from the Greek word “acantha,” meaning “thorn”) because of the spiked thorny appearance of red blood cells (De Franceschi et al., 2014; Adjobo-Hermans et al., 2015). In fact, neurons and erythroid progenitor cells in the bone marrow were recently shown to share common gene regulatory pathways defining their fate and properties (Kinney et al., 2019).

The heart and blood also have much in common (Kaestner, 2013). The mortality of patients with myocardial infarction (acute coronary syndrome) and those undergoing valve replacement surgery may be predicted based on the degree of variance in red blood cell shapes and sizes (Ghaffari et al., 2016; Duchnowski et al., 2017; Abrahan et al., 2018). Furthermore, red blood cells were recently shown to function as actors, not passive witnesses, in cardiovascular diseases, contributing to the regulation of redox state and vascular tone and activating protective or disruptive signaling cascades in the myocardium and blood vessels (Pernow et al., 2019). Can red blood cells be regarded as deputies for other organs of our body, such as the brain and the heart?

One more exciting and rapidly developing area aims at revolutionizing blood donations and transfusions. Instead of relying on people readily offering their blood for the others to use, researchers are producing, so far in very small amounts, red blood cells of the type needed for each individual patient in a test tube (Shah et al., 2014; Hawksworth et al., 2018). However, it will take some time until cell culture can upscale to provide enough red blood cells for transfusion. Therefore, before this happens, we still have to rely on blood donations and do our best to improve the red blood cell storage conditions (D’Alessandro and Seghatchian, 2017) and to manage and reduce damage of cells during lesions (Yoshida et al., 2019). Furthermore, each patient may decide in the future to use his/her own red blood cells as transport containers to deliver toxic drugs to the location in the body where they are supposed to act without poisoning the host (Villa et al., 2016; Sun et al., 2017). Nature itself has chosen to modify components of red blood cells to protect hosts from *Plasmodium* infection causing a deadly disease that claimed over 400 000 lives in 2018 alone, malaria (Weatherall, 2008; Timmann et al., 2012; Malaria Genomic Epidemiology et al., 2015). This evolutionary selection has taken ages to occur and may now be of use to the development of protective strategies for the human population, as the spread of Plasmodium further to the north will follow the increase in atmospheric temperatures.

The universe of red blood cells spreads far beyond the cells that function in *Homo sapiens*. In agreement with the Hitchhiker’s Guide, we have learned much about RBCs in the true rulers of the Earth, mice. These furry fellows give us a chance to study the mechanisms of diseases and to design new therapies for mice and humans. Our knowledge of the red blood cells in other species, including our pets and other tamed and wild, warm- and cold-blooded creatures that attend veterinary clinics from time to time, is rather fragmentary and requires more attention (Figure 1).

The ultimate “Answer to the Ultimate Question of Life, the Universe, and Everything” may only be given as we keep working and using our brains along with artificial intelligence. The next edition of “The Guide to Red Blood Cells” is on the way, and the motto for the early career scientists in the area stands as stated by Adams: “Don’t Panic.”

## Data Availability Statement

The original contributions presented in the study are included in the article/supplementary material, further inquiries can be directed to the corresponding authors.

## Author Contributions

All authors listed have made a substantial, direct and intellectual contribution to the work, and approved it for publication.

## Conflict of Interest

The authors declare that the research was conducted in the absence of any commercial or financial relationships that could be construed as a potential conflict of interest.
